# The Effect of Dehydration on Tooth Color: A Prospective In Vivo Study

**DOI:** 10.7759/cureus.48140

**Published:** 2023-11-02

**Authors:** Céline Alamé, Carina Mehanna Zogheib

**Affiliations:** 1 Department of Restorative and Esthetic Dentistry, Saint Joseph University, Beirut, LBN

**Keywords:** tooth, spectrophotometry, rubber dam, dehydration, color

## Abstract

Introduction

The influence of dehydration on tooth shade constitutes a significant aspect, making it a pivotal area of study, particularly within the field of restorative dentistry. This prospective study was done to analyze, using a spectrophotometer, how dehydration affects the shade of the teeth.

Materials and methods

Twenty-five participants were recruited in this prospective study that took place in the Dental Care Center of Saint Joseph University of Beirut (Lebanon) between October 2022 and January 2023. The inclusion criteria for this study primarily comprised four intact maxillary incisors. On the other hand, damage, cavities, restorations, and staining served as exclusion criteria.

The VITA Easyshade Advance (Vident, Brea, CA) spectrophotometer was used to record the measurements. The measures of L (which represents darkness to lightness), a (which represents greenness to redness), and b (which represents blueness to yellowness) were registered at the center of each tooth's labial surface at baseline and then at 10-minute intervals for 30 minutes while the teeth were dehydrating due to the placement of a rubber dam. The color difference (ΔE) was later calculated using L*a*b* measures, with the perceptibility threshold set at ΔE00=0.8. Statistical analyses were performed using the Friedman test, the Bonferroni post hoc test, and the Wilcoxon signed-rank test.

Results

The different color changes represented by ΔE (ΔE1, ΔE2, and ΔE3) were found to be significantly higher than the perceptibility threshold of 0.8 (p<0.001). A statistically significant difference was found between ΔE1 and ΔE3 (p<0.05). Moreover, a statistically significant difference was found between L0, L10, L20, and L30 (p<0.001). Statistically significant differences (p<0.05) exist between the means of L0 and L20, the means of L0 and L30, the means of L10 and L20, and the means of L10 and L30.

Conclusion

This study showed that dehydration affected the shade of the teeth: Lightness increased, and therefore, the teeth appeared whiter. Dentists should consider the hydration status of the teeth when evaluating color for treatments, as dehydration can significantly affect shade matching: the more the dehydration time elapses, the more the color difference compared to the baseline increases.

## Introduction

In dentistry, the shade of the teeth is an essential factor that determines the aesthetics, as well as the patient's satisfaction. Shade matching is therefore a crucial step that reflects the success of the aesthetic restoration outcome [1]. According to several dentists and technicians, this step represents the major challenge. Thus, particular importance must be given to it [2].

Depending on the practitioner's preference and dexterity, two methods can be used to measure shades: the visual method or the instrumental method.

The visual method consists of comparing a tooth to shade guide tabs to find the sample that most closely resembles the color of the adjacent teeth. Dental shade guides are based on Munsell's three dimensions of color, which are hue, chroma, and value [3]. Hue is the color of an object that differentiates one color family from another. Chroma is the intensity of the pigment or hue. Value describes the color on a white-to-black-gray scale, depending on the reflection of color. It is the most essential parameter in dentistry because the slightest difference can be easily detected than a difference in the other two parameters [4-7]. This inexpensive method is used by most practitioners due to its simplicity. However, it is subject to many errors and inaccuracies since the result is very subjective: it depends on the ocular physiology of the practitioner, as well as the quality of the surrounding light [8]. Hence, it is essential to acknowledge that this method has limitations that can impact the precision and dependability of the outcomes generated by this method. Some are observer-related limitations, which are associated with the individuals participating in the method. These limitations encompass factors such as age, gender, experience, the presence of fatigue, color vision deficiencies, and any influence introduced by medication use. Others are limitations stemming from the environment in which the method is conducted, such as the ambient lighting and external variables [3,4,9,10]. Therefore, the instrumental technique is slowly taking over this conventional method [2].

The instrumental method involves using an instrument that measures light energy reflected from an object in the visible spectrum to determine hue [11]. This expensive method was introduced to overcome the limits of the visual technique and is thus much more objective. Among the various categories of instruments present (spectrophotometers, colorimeters, and digital imaging devices), the spectrophotometer is the most used: it is the gold standard since it is one of the most accurate, quick, and objective instrumental methods used in dentistry for shade matching. The resulting color measurements can be linked to different dental shade guides and transformed into the appropriate shade tab [3,10,12]. However, only one point can be read at a time for the spectrophotometric evaluation [11,13].

Each of the three approaches (spectrophotometers, colorimeters, and digital imaging devices) can deliver different readings from a system of an instrumental color measurement developed by the Commission Internationale de l'Éclairage (CIE) called the CIE L*a*b* color space [14]. This system describes colors using the following three axes: L* value quantifies the lightness of an object on a scale from zero (pure black) to 100 (white), a* value measures the quantity of red (positive a*) or green (negative a*), and b* value measures the quantity of yellow (positive b*) or blue (negative b*) [6,14-16].

Combining both the instrumental technique and the visual technique is advised because they complement each other [3].

Practitioners cannot perceive shades similarly [1] because the shade is influenced by several extrinsic factors such as the restoration environment, the luminosity, the visual fatigue, and the subjectivity of the practitioner [11]. Moreover, it is also influenced by a major intrinsic factor, which is dehydration.

In dentistry, several procedures can cause tooth dehydration, such as rubber dam isolation, dental impressions, and tooth bleaching [1,17,18]. A study done by Russel et al. proved that the teeth appear lighter and less saturated when dehydrated by using a rubber dam. They later allowed the teeth to rehydrate: it took them 20 minutes to return to the color of baseline [1,19]. The same thing happened when they took impressions, but the teeth needed 30 minutes to return to the color of baseline [1]. Furthermore, during teeth bleaching, tooth dehydration exaggerates the bleaching effect and may lead to erroneous color change: At first, the patient is satisfied, but one week later, partial color rebound is noted due to rehydration [17,18].

The layers of the natural teeth that affect shade must be understood to comprehend how hydration affects shade matching. The chroma of a tooth is primarily determined by the dentin layer. Through light scattering that can be determined by refractive indexes, the enamel layer alters the appearance of the chromatic dentin layer [20]. When dehydrated, the teeth appear whiter because dehydration increases the opacity of the enamel. Air replaces the water that was previously present in the inter-prism spaces between hydroxyapatite crystals of the enamel, and thus, the refractive index changes. Therefore, light scattering is reduced, causing the enamel to appear opaque and white, masking the dentin's chromatic layer. Hence, the translucency will decrease, and the luminosity will increase [1,18,20-23].

According to Hatırlı et al., it is important to choose the shade before any dehydration, that is, before performing any restoration and especially before the installation of the rubber dam [18].

The primary aim of this study was to evaluate the effect of dehydration on the shade of the teeth. The null hypotheses tested were the following: (1) The shade of the teeth does not vary according to their degree of hydration, and (2) the ability of shade matching could be conducted at any given time.

## Materials and methods

Setting

This is a prospective in vitro study, conducted in in the Dental Care Center of Saint Joseph University of Beirut (Lebanon) between October 2022 and January 2023, after the approval of the Research Ethics Committee of Saint Joseph University (Tfemd/2023/46). A consent form was then signed by all the participants before taking the measurements.

Participants

In this study, a total of 25 fourth- and fifth-year bachelor's students of the Faculty of Dental Medicine were recruited, based on a sample size calculation. To determine it, a power analysis was conducted, with the change in color (ΔE) serving as the primary outcome. The analysis utilized a significance level (α) of 5% and a power level (β) of 5%, corresponding to a power of 95% and an effect size of 0.25. The initial estimated sample size was 50 teeth, adjusted for a 15% additional estimate. The sample size calculation was conducted using G*Power Version 3.1.9.2 (Heinrich Heine University Düsseldorf, Düsseldorf, Germany).

Only participants meeting the inclusion criteria participated in this study: all participants selected had four intact maxillary incisors. These teeth did not present any damage, cavities, restorations, or staining.

However, the teeth that were bleached in the previous year and presenting brackets or palatal fixed retainers were excluded from this study.

In this study, the presence or absence of tobacco or alcohol habits among the patients was not taken into consideration.

Variable

The variable is the variation of the shade of the teeth, linked to the degree of dehydration and the time elapsed.

Data sources and measurement

**Figure 1:**
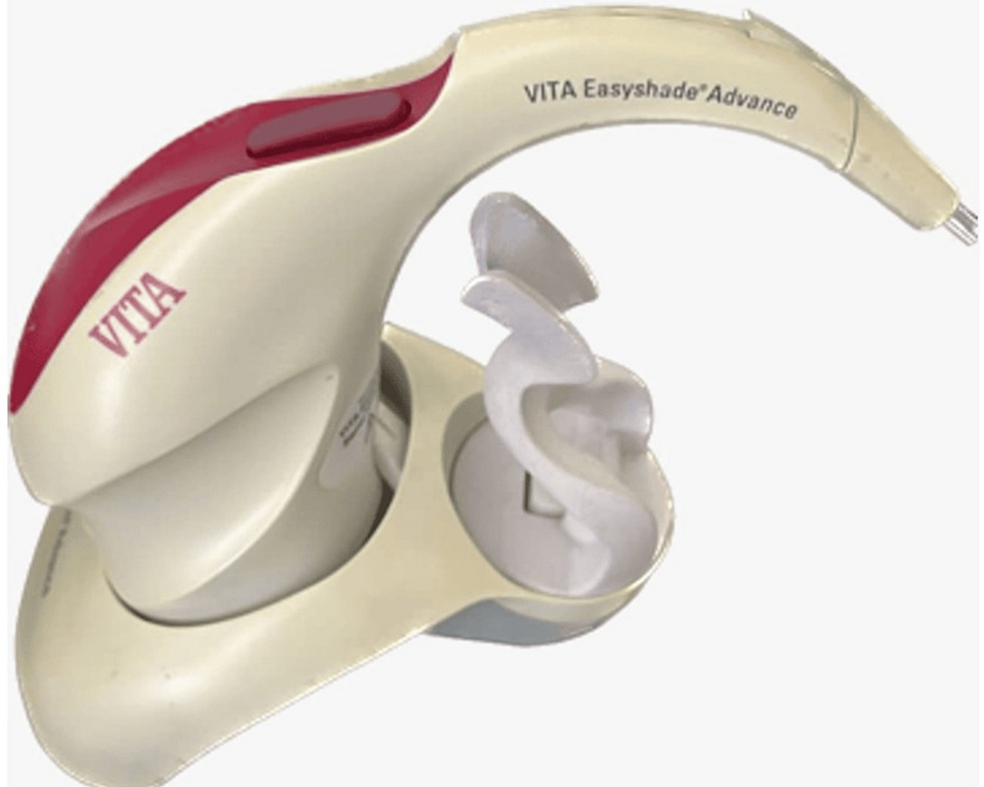
VITA Easyshade Advance (Vident, Brea, CA) spectrophotometer

The VITA Easyshade Advance (Vident, Brea, CA) spectrophotometer (Figure 1), one of the most precise and frequently used devices [24], was used to collect the data. To be able to measure the color of the teeth, the spectrophotometer had to be calibrated first by placing the probe tip in the calibration port aperture. The instrument was then put at the center of the labial surface of each tooth to register the L*a*b* measurements at different timings. The first measurement was taken at baseline, before any dehydration. Next, the teeth were dehydrated by placing a rubber dam without blowing any air. The second, third, and fourth measurements were performed at 10-minute intervals for 30 minutes: 10, 20, and 30 minutes.

In order to minimize the potential source of errors and to ensure consistency in data collection, all measurements were conducted by a single individual.

All the data collected was then registered in a table in Excel (Microsoft® Corp., Redmond, WA), and they were later used to calculate the degree of color difference (ΔE) using the following formula: ΔE=[(L*_1_-L*_2_)^2^+(a*_1_-a*_2_)^2^+(b*_1_-b*_2_)^2^]^0.5^ [25], where numbers 1 and 2 represented the various times of dehydration. For example, for ΔE1, which is the color difference between baseline and 10 minutes, 1 represented the measure taken at 10 minutes of dehydration, and 2 represented the measure taken at baseline (before any dehydration).

This formula is being used more frequently in dentistry for color research because it provides variables that can be compared using statistical tests. It is therefore the more suitable approach for scientific purposes [6,16,25].

Statistical methods

A descriptive analysis was carried out: each quantitative variable is described by its minimum, maximum, mean, and standard deviation (SD). 

The Friedman test followed by the Bonferroni post hoc test for pairwise comparisons was used to assess potential statistically significant differences in color changes between the different measures (at baseline, t=10 minutes, t=20 minutes, and t=30 minutes) and potential statistically significant differences in the L measures at baseline, t=10 minutes, t=20 minutes, and t=30 minutes.

The Wilcoxon signed-rank test was used to compare color variation to the perceptibility threshold (ΔE00=0.8) [18]. A p-value of less than 0.05 is considered statistically significant. The analysis was conducted using the Statistical Package for Social Sciences (SPSS) version 25 software (IBM SPSS Statistics, Armonk, NY).

## Results

After receiving a message outlining the inclusion and exclusion criteria, all participants voluntarily opted to participate, with the first 25 patients being selected, resulting in a sample of 100 teeth (n=100).

Variations of color difference measures

As evidenced in Table 1, ΔE1 has a mean of 7.06 (SD=4.05), ΔE2 has a mean of 7.95 (SD=4.42), and ΔE3 has a mean of 8.96 (SD=4.71). As for the differences between the different measures, a statistically significant difference was found between ΔE1 and ΔE3 (p<0.05).

**Table 1:** Color changes between baseline, t=10 minutes, t=20 minutes, and t=30 minutes (n=100) ^a^Friedman test. ^b^Significant difference using the Bonferroni correction for multiple tests (p<0.007) ΔE1, color difference between baseline and 10 minutes; ΔE2, color difference between baseline and 20 minutes; ΔE3, color difference between baseline and 30 minutes; SD, standard deviation

	Minimum	Maximum	Mean	SD	p-value
ΔE1	0.81	19.06	7.06^b^	4.05	0.007^a^
ΔE2	0.83	25.36	7.95	4.42	0.007^a^
ΔE3	1.04	23.35	8.96^b^	4.71	0.007^a^

**Figure 2:**
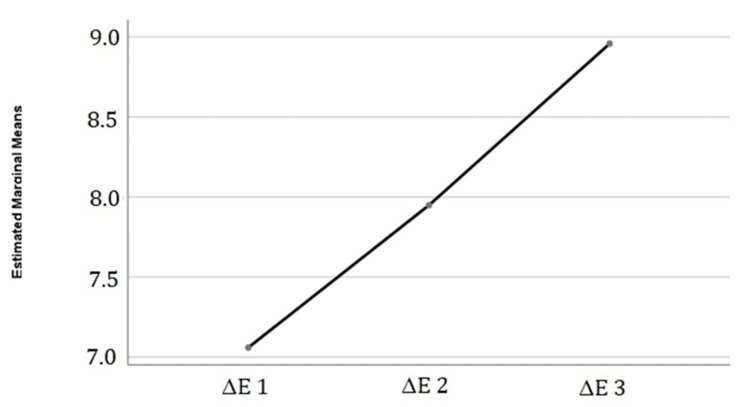
Color difference (ΔE) mean variations throughout time

Moreover, the different color changes (ΔE1, ΔE2, and ΔE3) were found to be significantly higher than the perceptibility threshold of 0.8 (p<0.001) (Figure 2).

Changes in the lightness measures

As evidenced in Table 2, L0 has a mean of 89.10 (SD=5.47), L10 has a mean of 90.52 (SD=5.29), L20 has a mean of 91.60 (SD=5.56), and L30 has a mean of 92.49 (SD=5.71).

**Table 2:** L measure at baseline, t=10 minutes, t=20 minutes, and t=30 minutes (n=100) ^a^Friedman test. ^b,c,d,e^Significant difference using the Bonferroni correction for multiple tests (p<0.001). The same lower-case letters show statistically significant differences between the pairs SD: standard deviation

	Minimum	Maximum	Mean	SD	p-value
L0	73.50	100.00	89.10^b,c^	5.47	<0.001^a^
L10	75.70	100.00	90.52^d,e^	5.29	<0.001^a^
L20	71.20	100.00	91.60^b,d^	5.56	<0.001^a^
L30	68.40	100.00	92.49^c,e^	5.71	<0.001^a^

The Friedman test revealed a statistically significant difference between L0, L10, L20, and L30 (p<0.001). The Bonferroni test suggests that statistically significant differences (p<0.05) exist between the means of L0 and L20, the means of L0 and L30, the means of L10 and L20, and the means of L10 and L30.

## Discussion

A major factor that may poorly affect the results of restorations is color change, which is primarily caused by tooth dehydration. Moreover, when coupled with office bleaching, tooth dehydration leads to a color change that occurs directly after treatment [18]. The principal aim of this study was thus to evaluate the in vivo effects of dehydration on the shade of the teeth and therefore deduce if color matching could be made at any time of the dental procedure.

The three different dehydration times of the study were based on several articles, as well as the clinical time needed for isolation [1,18,19].

To assess the clinical significance of the color change, ΔE measurements were calculated respectively: ΔE1 between baseline and 10 minutes, ΔE2 between baseline and 20 minutes, and ΔE3 between baseline and 30 minutes. All measures were compared to the perceptibility threshold. The perceptibility threshold, defined as the smallest color difference that can be detected by an observer, differs from one study to another: In some articles, this value was set at 1.5 [2], whereas for others, it was defined at 0.72 [22].

The benchmark for the perceptibility threshold used in this study was defined at 0.8. This reference is justified by a report from Paravina et al. in which the same value was said to have been used as the perceptibility threshold in half the studies on color change [18].

All three ΔE color changes (ΔE1, ΔE2, and ΔE3) of this study gradually increased and were found to be significantly higher than 0.8. This confirms that the color difference was perceptible by the naked eye at the three different intervals of dehydration. Our finding, which indicates that ΔE1 exceeds the perceptibility threshold, is consistent with the results reported by Ruiz-López et al. [22], who stated that "variations in color (color differences) and WI_D_ (whiteness index for dentistry) index variations exceeded their corresponding thresholds of perceptibility after short-term dehydration of the teeth (10 minutes)," and with the findings of Hatırlı et al. [18] who noted that "the evaluated teeth in the present study exhibited significant color change after 10 minutes of dehydration."

A statistically significant difference was found between ΔE1 (baseline and 10 minutes) and ΔE3 (baseline and 30 minutes). This result matches the findings of the study of Burki et al., who said that the color coordinate changed significantly between baseline and 10 minutes and baseline and 30 minutes [1]. This finding is valuable for practitioners whose procedures extend for a long time (30 minutes or beyond), as it underscores the importance of conducting shade matching prior to these procedures to ensure accurate color matching and prevent discrepancies between the chosen color and the color of baseline.

L*, a coordinate that represents lightness from a scale of 0 to 100, increased throughout the dehydration procedure. This result aligns with the findings of Du et al., even though their dehydration times differed from our study. In their research, they noted the statement that "after two-hour and four-hour dehydration, the value of L* had increased" [2].

**Figure 3:**
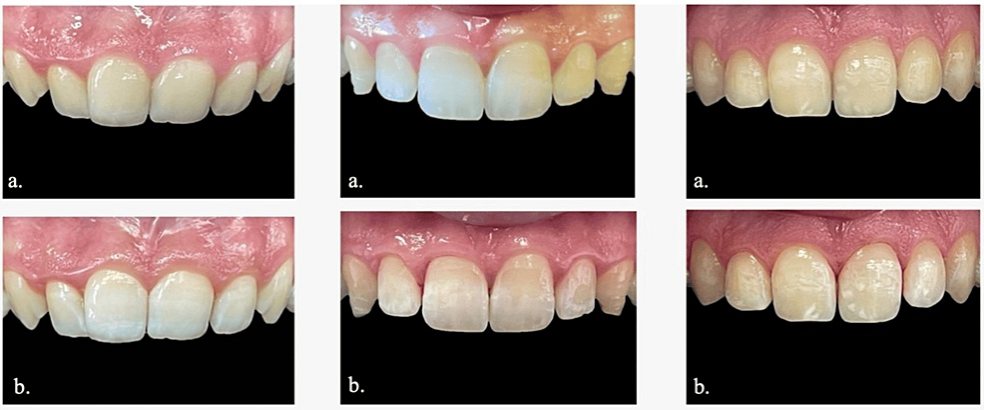
Photographs of the teeth before and after dehydration The photographs compare the teeth before rubber dam isolation (Figure 3a) and after 30 minutes of dehydration (Figure 3b). They clearly show a variation in tooth color after dehydration

In our study, we found that there is a statistically significant difference between L0, L10, L20, and L30. This difference is apparent between the means of L0 and L20, as well as between those of L0 and L30. In fact, the increased reflection of light may explain the increase in L* value. Because of dehydration, the interprismatic space is filled with air instead of water, which will alter the refractive indexes of the enamel. The refractive index will decrease from 1.33 (refractive index of the water) to 1 (refractive index of the air): this decrease in the refractive index will increase the reflection of light and therefore lead to a decrease of the transparency and an increase of both the lightness and the opacity of the enamel, thus resulting in whiter teeth (Figure 3) [2,26-28].

The limitations of this study include the repositioning error of the spectrophotometer. Some studies have recommended the use of a custom-made jig tailored for each subject to position the probe of the spectrophotometer more precisely during the different measurements [23]. In addition, the number of subjects who participated in this study might have affected the result, as the sample size was limited. The same applies to the number of teeth examined in each subject. An interesting approach would have been to increase the number of participants and decrease the number of teeth for each participant from four to two to further diversify the results. Another limitation was that all participants were at the beginning of their 20s. Since age and mineral contents can affect teeth color, an expansion of the age range may have likewise allowed different results [22,29].

A future study will be conducted to complement this research. Its objective will be to evaluate the time required to regain the teeth's color of baseline in the rehydration procedure.

## Conclusions

Based on these findings and on the limitations of this study, it was concluded that teeth dehydration significantly impacts teeth shade, particularly affecting the perception of whiteness. The duration of dehydration time directly corresponds to the extent of color difference from baseline. This strongly refutes our initial hypotheses, asserting that teeth shade remains constant regardless of hydration status and that shade matching can be indiscriminately performed at any time during a dental procedure.

These results emphasize the importance of carrying out shade matching prior to any procedure leading to teeth dehydration, such as rubber dam isolation. We hope that these insights will guide dental practitioners in enhancing their shade-matching practices, ultimately improving patient satisfaction. Further studies might focus on identifying ways to mitigate the impact of dehydration on color change or developing more adaptive shade-matching techniques that account for teeth hydration levels.
